# The Use of Chloroform in Dental Operations

**Published:** 1879-04

**Authors:** 


### ARTICLE II.

### *The Use of Chloroform in Dental Operations.*

Extract from Proceedings of the Odontological Society of Great Britain, November 4, 1878.

The President proceeded to open the discussion on "The Use of Chloroform in Dental Operations." He began by reminding those present that the discussion had been suggested by the editor of the *British Medical Journal.* In commenting on the death of a child in May of this year, from the effects of chloroform, administered to facilitate a dental operation, he concluded his remarks on the case with the following query: "Is it ever right to give chloroform for dental purposes? We wish the Odontological Society would pronounce an authoritative opinion on the subject, and we should hope that if they were to do so they would absolutely forbid it." It was well known that the editor of the *British Medical Journal* had for a long time past taken a deep interest in the subjects of deaths from chloroform, and that he had repeatedly expressed a very decided opinion as to the danger and responsibility attached to its use. As the result of this deliberately formed conviction, he had called upon the society to express its opinion on a practical question of the utmost importance, and he (the President) thought that they ought to respond to the call. If, as the writer of the article implied, the use of chloroform for dental operations was really unnecessary, then undoubtedly those who continue to employ an agent whose use might be attended with such terrible consequences incurred a most serious responsibility. There were several subsidiary points of great interest which might be discussed, but, as it was important to endeavor to arrive at some definite answer to the question he had quoted, he thought it would be better to confine the discussion to it in the first instance, viz: "Is it ever justifiable to administer chloroform as an anæsthetic for dental operations?"

Mr. Clover, unfortunately, was unable to be present, but had expressed his opinion in a letter. He was, however, pleased to see several gentlemen present who had had great experience in the use of anæsthetics, and he hoped that they would favor the society with their opinions. Mr. Clover's answer to the question was as follows: "I think chloroform may be justifiably given in cases of dental operation in which complete quietude of the patient is necessary for more than two minutes. Ether would be somewhat less dangerous, but it is very difficult to keep the patient absolutely quiet, and the bleeding is considerably increased when ether is used."

Mr. Browne Mason, of Exeter, had also written to the Secretary as follows:

"To this question, Is it justifiable to administer chloroform for dental operations? I would say that if anæsthesia by chloroform is justifiable at all, it is as much so in dental operations as in any other. Nitrous oxide gas does not answer for prolonged operations by reason of the transitory nature of its effects, so there is nothing left except ether or chloroform. I have seen more of chloroform than of ether, and I never witnessed any untoward symptom from its use in a practice extending over twenty years; but, as ether is undoubtedly the safer, I think the preference should be given to it when prolonged anæsthesia is required."

The President then called upon Mr. Woodhouse Braine, who said he quite agreed with Mr. Clover's answer to the question; he considered that the use of chloroform was perfectly justifiable in cases where a safer anæsthetic was not applicable, as in prolonged operations. It was true that chloroform occasionally caused fatal results, but the diseases of the mouth which were sufficiently serious to require the use of chloroform might themselves be fatal; such operations were not mere matters of expediency. Mr. Braine then went on to report the death from chloroform of a child at the unusual age of two years and four months, which had occurred a few days previously.

Mr. John Tomes said he had had considerable experience with both chloroform and ether; he had found them both very useful, and had never witnessed any ill effects from either of them; under these circumstances, he did not feel disposed to turn round on old friends and condemn them.

Mr. Bailey thought that Mr. Clover's acknowledged high position as an authority on the use of anæsthetics entitled his opinion to great weight. His own opinion was that gas was the most suitable anæsthetic for dental operations, and that if gas was not available, ether was better than chloroform, but that if prolonged insensibility was required, chloroform must be resorted to, in spite of the fact that death occasionally resulted from its use.

Mr. Henry Barrett said he had not come to the meeting with any intention of joining in the discussion, but as the President seemed anxious to hear his experience of anæsthetics, he would state it briefly. About the year 1846 dentists began to be called upon to use ether for dental operations; he did so with others, and continued to give ether with safety to his patients and satisfaction to himself for some years. Suddenly it was found that ether was dangerous, and chloroform was had recourse to; chloroform was given for some fifteen years without any trouble or apparent danger, when all at once it was discovered to be more dangerous than ether, and one authority went so far as to say that any one who continued to administer chloroform in spite of the evidence which had been collected against it, ought, in the event of fatal result, to be committed by the coroner on a charge of manslaughter; but, notwithstanding all the strong language which had been indulged in, he personally had never seen any danger; and, although he advocated the utmost care and caution in the use of chloroform, he did not feel disposed to condemn it altogether.

Mr. Jonathan Hutchinson said he could from personal experience testify to the superiority of nitrous oxide gas over other anæsthetics. He would allow any one who had had some experience in the use of the gas to give it to him, but he would only take ether from a thoroughly qualified person, and would not allow any man living to give him chloroform. He had used both ether and chloroform largely; he had lost one patient from chloroform eighteen years ago, and had had many alarming cases; he had seen fatal cases also under the care of others. During the last six years he had almost invariably used ether, and during that time he had not had a single alarming case. He strongly disapproved of some of the complicated inhalers now in use, in which, for the sake of economizing ether, the patient was made to reinhale his own breath, thus taking in a quantity of carbonic acid as well as ether. In the only bad cases connected with ether which he had seen some such apparatus had been used. He greatly preferred a simple leather mouth-piece, perforated at the top, with a sponge and towel inside; in this way the patient got plenty of air and plenty of ether. There certainly were a few cases in which chloroform was preferable, viz: for old people and very young children. Old people over sixty did not always recover readily from ether narcosis. He had met with several cases in which there had been alarming continuance of insensibility, and in one case death seemed to be partly due to this cause. Chloroform seemed to be less dangerous than ether to old people; and in the case of infants there seemed to be practically no danger at all, while it was most dangerous to young people. At the hospital in Moorfields there used to be on an average a death from chloroform every year, and they were nearly always young people operated upon for strabismus. Chloroform was certainly more convenient in some respects and pleasanter to take than ether; patients who had tried both almost invariably preferred the former; but he thought it criminal to place a patient in danger of losing his life by giving chloroform, when ether had been found to be so much safer.

The President asked Mr. Hutchinson whether he did not think that dental operations presented some peculiar features which entitled them to special consideration?

Mr. Hutchinson said he could not call to mind any points in which dental operations differed materially from those in general surgery; he thought that the opinions he had expressed would apply equally well to them.

Mr. Mills said that his answer to the question would be "Yes, in certain cases." At the same time he did not consider chloroform to be the most suitable anæsthetic for dental operations generally. Nitrous oxide was the best in the majority of cases, and when more prolonged insensibility was required than could be obtained by the use of the gas, he thought the next best plan was to supplement this by ether; gas and ether inhalatio nwas not attended by the danger of syncope, which was so great a cause of anxiety in giving chloroform. At the same time there were undoubtedly cases in which chloroform was the best agent; it was best for old people, especially if they had any tendency to bronchitis, and it was best for very long operations. Although he had seen more bad cases from chloroform than from either gas or ether, and admitted that it required more care in its administration, still he thought its use was justifiable in certain cases.

Mr. Braine said he wished to call Mr. Hutchinson's attention to some important points in which dental operations differed from those in ordinary surgery in regard to the use of anæsthetics. In the first place, the mouth had to be kept wide open, and had also to be left as free as possible for the use of the operator; it was, therefore, very difficult to regulate the amount of air which the patient inhaled. When the patient was once under the influence of chloroform, insensibility could be kept up by the aid of the nose-piece, but to keep a patient under the influence of ether without at the same time hindering the operator was a difficult matter. Then again, ether caused profuse secretion of saliva, and greatly aggravated any hemorrhage which might occur, and the collection of blood and saliva was often an annoyance to the dentist by obscuring the parts. These were some of the objections which might be urged against the use of ether for dental purposes.

Mr. Charles S. Tomes said he would state, as briefly as possible, what his practice was; his opinions might then be readily inferred. He would never again allow a patient to take chloroform or a doctor to give it at his house, nor would he, under any circumstances, advise a patient to take chloroform; but if the patient wished to do so, and he thought that the case justified its use—such cases were very rare—he would insist on the patient taking it in bed at his own house, with all the precautions which were usual before the performance of other surgical operations. Given in this way, there would not only be less risk to the patient but in the event of a fatal accident, no blame could attach to the dentist. With reference to what has been said as to the unsuitability of ether for dental purposes, he could only say that he had seen several cases of cleft palate successfully operated on under ether at a New York hospital, and that the patients were kept well under its influence without in the least impeding the movements of the surgeon; all danger and trouble from bleeding was obviated by laying the patient on his side, so that the blood ran into the cheek and out at the angle of the mouth. After this evidence of what was possible, he might be pardoned for thinking that the adaptation of ether to dental requirements was merely a question of management.

Mr. Bird thought that the use of any kind of anæsthetic in dental operations might generally be looked upon rather as a luxury than as a necessity. Nitrous oxide was certainly the most suitable for dental practice; even somewhat lengthy operations could be completed by means of successive administrations. He thought that it had been proved that the sitting posture usual in dental operations increased the risk attached to chloroform inhalation; he, therefore, quite agreed with the precautions which Mr. Charles Tomes had suggested.

Mr. Woodhouse said he would adopt Mr. Tomes's plan, and state his usual practice with regard to anæsthetics. He used nitrous oxide in all cases where it could be made available. Formerly, in cases where a number of teeth or stumps had to be removed, he used to give chloroform, and extract all of them at one sitting; now he used the gas, and had it administered a second, third, and, if necessary, a fourth time, on different days, extracting two or three on each occasion. Still, cases did occur in which nitrous oxide was useless, on account of the brief duration of the insensibility, and he considered that, under these circumstances, it was just as justifiable to give chloroform for a dental as for any other surgical operation.

Mr. Underwood said that during the eight years following the introduction of nitrous oxide, chloroform had not once been given in that hospital. In his private practice, he had during the same period used chloroform only three times. He thought that these facts proved that, though it was impossible to say that the use of chloroform was never justifiable in dental practice, still that the cases in which it was required were very rare. In the great majority of cases nitrous oxide would suffice. He agreed with Mr. Woodhouse that there was no object in extracting a large number of teeth at one sitting. His opinion was that chloroform should be reserved for very exceptional cases, and practically he found that it was very seldom required.

Mr. Pedley said that, after hearing the use of chloroform in dental practice condemned by such high authorities, he could not help feeling somewhat guilty, for he had given it twice during the past two months. And yet, on consideration, he thought what he had done was justifiable; both were cases of impacted wisdom teeth, and their elevation and extraction was a very troublesome business, occupying in each case about a quarter of an hour. Gas was, of course, quite useless, and he personally found it very difficult to keep a patient under the influence of ether with his mouth gagged wide open, as was necessary in such cases as these. It might be a long time before he again met with a similar case; but having so recently experienced the value of chloroform, he did not feel disposed to answer the President's question with an unconditional negative.

Mr. Hunt said that at one time he was in the habit of giving chloroform four or five times a day, but for the last ten years he had used nitrous oxide almost exclusively, and had only had twelve chloroform cases during the whole of that time. He had never seen a death from chloroform, though he had seen several alarming cases; he had seen also one narrow escape from death by nitrous oxide. Ether might be somewhat less dangerous than chloroform, but it was certainly difficult to manage when given for operations in the mouth. His experience was that nitrous oxide would do in the great majority of cases, but that there were cases in which the use of chloroform was necessary, and he thought that its use under these circumstances was justifiable.

Mr. Sewell said that toward the end of his studentship at St. Mary's Hospital two patients died under chloroform; in both cases the operation which was being performed was a trivial one, and in neither could any disease be found to account for death. These deaths occuring as he was starting in practice, made a deep impression on his mind, and this impression was strengthened by the arguments of Dr. B. W. Richardson, with whom he happened at that time to come in contact. He was in the habit of using gas constantly, and up to that time had not met with a case in which, according to his ideas, the use of chloroform was justifiable. Such a case might occur, and should he ever meet with one he should certainly adopt the precautions which had been suggested by Mr. Charles Tomes.

The President said that all who had spoken had agreed that the use of chloroform in dental operations could not be absolutely forbidden, but that it should be restricted to very exceptional cases. Mr. Charles Tomes had added some very practical suggestions; he said it should only be given at the patient's own house and with the patient in a recumbent position. His own opinion was that a previous consultation with the medical attendant of the patient was also desirable. He proposed that the Society should adopt these suggestions in the form of a resolution, thus: "That it is the opinion of the Society that the use of chloroform in dental practice should be restricted to very exceptional cases, that it should only be given at the patient's own house and with the patient in a recumbent position, and that whenever circumstances will admit of it a previous consultation with a qualified medical practitioner is highly desirable."

Mr. Dennant thought that, with all deference to the President, the adoption of any such resolution was very undesirable. In London it would be easy enough to comply with its conditions; any of the members present could at any time obtain the assistance of experts thoroughly skilled in the use of the anæsthetic which might be deemed most suitable for the case. But dentists residing in the country had only country surgeons to depend upon, and they were obliged to conform to their usages. Gas and ether had been for some years before the profession, but their use was not yet general throughout the country; in many parts chloroform was still the only anæsthetic with which the medical practitioner was familiar. It would not be fair to pass the resolution in the name of the Society. The meeting was not a truly representative one; it was composed almost entirely of men residing in or near London, while the bulk of the members were scattered throughout the length and breadth of the kingdom, and, as he had shown, these would be the people who would be chiefly affected by the resolution.

Mr. John Tomes could not see why the responsibility of answering this question should be thrust upon the dental profession only; it was one for the whole medical profession to decide, and its terms should not be is chloroform justifiable in dental operations, but in minor surgical operations generally. The question had been fairly discussed, and he thought they should be contented with the general agreement of opinion which had been expressed.

Mr. Walker said he agreed with Mr. Braine and other speakers, that chloroform was preferable to ether for dental operations. He had given ether a fair trial, but the result had not been satisfactory. He was in the habit of using chloroform occasionally, but he always placed the patient in the recumbent position, unloosed every band, and generally observed the same precautions as if the operation were a capital one.

Mr. Charles Tomes thought that the passing of a resolution without notice was an unusual termination to a scientific discussion, and might possibly become a bad precedent. He doubted whether the majority of the members present had given sufficient attention to the question, or had had sufficient evidence before them to enable them to decide it as carefully as its importance deserved; he would advise that the resolution be not passed.

The President said he should be sorry to urge the Society to come to a definite conclusion against their inclinations. No doubt practitioners in London and other large towns were more favorably situated as regarded the facility of obtaining advice and assistance than were their brethren in the country, and it was possible that an unfair use might occasionally be made of the resolution by the legal profession. He would not, therefore, press it. It was the less necessary since all the speakers had agreed that though the use of chloroform might be occasionally justifiable, it was very undesirable to use it in any case where the services of other and safer anæsthetics could be made available, and he felt sure that the strong conviction of the danger and responsibility attached to the use of chloroform which had been expressed by some of the speakers would have great influence with the profession.—*British Journal of Dental Science.*

